# The transcriptional atlas, intercellular communication, and metabolic reprogramming of the cartilage and synovium in osteoarthritis patients with diabetes mellitus: a multiomics study

**DOI:** 10.1302/2046-3758.156.BJR-2025-0474.R1

**Published:** 2026-06-01

**Authors:** Yinxian Wen, Siyi Liu, Haitao Chen, Ming Tu, Danyang Zhao, Junwei Xing, Wanqin Zheng, Yongkang Zhong, Hui Wang, Liaobin Chen

**Affiliations:** 1 Division of Joint Surgery and Sports Medicine, Department of Orthopedic Surgery, Zhongnan Hospital of Wuhan University, Wuhan, China; 2 Joint Disease Research Center of Wuhan University, Wuhan, China; 3 Department of Pharmacology, Basic Medical Science of Wuhan University, Wuhan, China; 4 Hubei Provincial Key Laboratory of Developmentally Originated Disease, Wuhan, China

**Keywords:** Osteoarthritis, Diabetes mellitus, Multiomics, Osteoarthritis (OA), cartilage tissues, chondrocytes, synoviocytes, RNA, fibroblasts, amino acids, pathogenesis, fibroblast growth factors

## Abstract

**Aims:**

A strong correlation has been observed between diabetes mellitus (DM) with the pathogenesis of osteoarthritis (OA). This study aimed to elucidate the cellular and molecular mechanisms by which DM exacerbates OA, through multiomics analysis of synovium and cartilage from OA patients with type 2 DM (T2DMOA).

**Methods:**

Single-cell RNA sequencing, bulk RNA sequencing, and metabolome profiling were performed on knee cartilage and synovium from 21 patients with OA or T2DMOA to investigate the differences in transcriptional landscape, intercellular signalling networks, transcription factor regulatory patterns, and alterations in metabolic pathways.

**Results:**

Single-cell profiling of synovium-cartilage tissues revealed distinct pathological alterations in T2DMOA patients compared to non-diabetic OA controls. In the T2DMOA synovium, we observed significantly enhanced differentiation of sublining fibroblasts into lining fibroblasts, along with enriched pathways governing energy metabolism, vasculogenesis, and cell proliferation. For cell-cell communication between cartilage and synovium, hepatocyte growth factor (*HGF*)-mesenchymal-epithelial transition factor (*MET*), fibroblast growth factors (*FGF*) 10-FGF receptor 1 (*FGFR1*), and nicotinamide phosphoribosyl transferase (*NAMPT*)-(integrin subunit alpha 5 (*ITGA5*) + integrin subunit beta 1 (*ITGB1*)) from synoviocytes to chondrocytes, as well as angiopoietin-like protein 2 (*ANGPTL2*)-toll-like receptor 4 (*TLR4*) and *ANGPTL2-*(*ITGA5 + IGTB1*) from chondrocytes to synoviocytes, showed a notable increase in T2DMOA patients. Conversely, T2DMOA cartilage exhibited a pronounced suppression of metabolic activity, particularly in amino acid transport and glycan biosynthesis. Additionally, transcription factors of synoviocytes and chondrocytes were clustered into five and four major modules, respectively, with various functions.

**Conclusion:**

Our findings define a diabetes-specific OA phenotype, characterized by aberrant synovial fibroblast activation, dysregulated synovium-cartilage crosstalk, and impaired cartilage metabolism. This integrated view establishes T2DMOA as a unique metabolic-subtype of OA, driven by disrupted intercellular communication and metabolic reprogramming.

Cite this article: *Bone Joint Res* 2026;15(6):584–600.

## Article focus

- Multiomics profiling of transcriptomic and metabolic landscapes in cartilage and synovium from type 2 diabetes mellitus osteoarthritis (T2DMOA) patients.
- Investigate the differences in transcriptional landscape, intercellular signalling networks, transcription factor regulatory patterns, and alterations in metabolic pathways on knee cartilage and synovium from patients with OA and T2DMOA using single-cell RNA sequencing.

## Key messages

- Transcriptomic data showed that the differentiation of fibroblasts into lining fibroblasts was significantly increased in T2DMOA, and the energy metabolism patterns of chondrocytes and synoviocytes shifted.
- For the cell-cell communication, angiopoietin-like protein 2 (ANGPTL), macrophage migration inhibitory factor (MIF), cyclophilins (Cyp) A, nicotinamide phosphoribosyltransferase (NAMPT), and fibroblast growth factors (FGF) signalling acted as the major messengers from synoviocytes to chondrocytes, with MIF, secreted phosphoprotein 1 (SPP1), FGF, CypA, and annexin from chondrocytes to synoviocytes.
- The sulphur metabolism pathway and metabolites in the synovium and pathways and metabolites in energy metabolism, amino acid metabolism and transport, and glycan metabolism in cartilage were altered in T2DMOA.

## Strengths and limitations

- This study has provided insights into the transcriptional landscape, intercellular signalling networks, cellular metabolic alterations, and gene regulatory networks in the pathogenesis of T2DMOA.
- Although we used four paired samples of articular cartilage and synovium, small sample size of each group still remains a major limitation.
- These preliminary findings warrant further experimental validation to establish their biological and clinical significance.

## Introduction

Osteoarthritis (OA) is now the most prevalent joint disease, as well as the major cause of joint pain and disability in the elderly, with 527.81 million global and 132.81 million Chinese cases in 2019.^1^ Diabetes mellitus (DM) is another major public health concern worldwide, with a prevalence of 9.3% globally and 9.7% in the USA.^2^ Accumulating clinical data have indicated a strong correlation between OA and multiple metabolic disorders including type 2 DM (T2DM).^3,4^ A prospective study demonstrated that knee OA incidence is significantly associated with DM (hazard ratio (HR) = 1.29; 95% CI 1.02 to 1.78), bad glycemic regulation (HR = 1.41; 95% CI 1.05 to 2.09), and long-term DM (≥ 5 years; HR = 1.49; 95% CI 1.02 to 2.17).^5^ T2DM was also recognized as a predictor of joint space reduction in males with established knee OA.^6^ Furthermore, DM is associated with an elevated risk of postoperative complications following joint arthroplasty.^7,8^ Other experimental studies have identified that DM could serve as an independent risk factor for the pathogenesis of OA.^9-11^ DM could aggravate or even trigger the progression of OA of the knee, as DM-caused systematic metabolic disturbances and metabolic rewiring of chondrocyte itself are both critical parts of OA pathogenesis.^9-11^ Such findings have raised the concept of diabetic OA, a novel sub-type of OA, and the question of how the two major disorders interact with each other.

The articular cartilage and synovium, the two major components of the joint, communicate with each other through the synovial fluid to accomplish substance exchange and signal transduction. The articular cartilage is a relatively avascular and homogeneous tissue with limited cell types of chondrocytes, chondroblasts, and few progenitor cells, of which the chondrocytes are the most predominant cell type.^12,13^ The synovium consists of a continuous surface layer and the underlying tissue, containing multiple sub-populations, including macrophages, fibroblast-like synoviocytes, lymphocytes, and endothelial cells, which act as the main barrier to the transport of molecules from plasma to synovial joint and a major modulator of cartilage homeostasis.^14^ Communication among chondrocytes and synoviocytes through substance exchange and signal transduction is believed to be critical to the maintenance of the homeostasis of the joint.^15,16^ As DM status reprogrammes the metabolism of such cells,^11,17,18^ essential differences may exist between the T2DMOA and ordinary OA. However, the cellular interplay between the articular cartilage and synovium, cell-cell communication among chondrocytes and synoviocytes, and such essential differences remain unclear.

Single-cell RNA sequencing (scRNA-seq) is frequently employed to delineate cellular and molecular linkages between distinct disease.^19-21^ Here, we have performed a multiomics study and data integration based on scRNA-seq, bulk RNA sequencing (RNA-seq), and metabolome profiling to investigate the transcriptional landscape, intercellular signalling networks, cellular metabolic alterations, and gene regulatory networks in patients with OA or T2DMOA. Our findings offer an unbiased atlas of the synovium and articular cartilage of T2DM patients with OA (T2DMOA) and provide new insights into T2DMOA, a novel subtype of OA.

## Methods

### Participant recruitment

Human articular cartilage and synovial tissues were obtained from 21 patients with knee OA (12 non-diabetic and nine with T2DM, aged between 49 and 83 years, grade 4 according to the Kellgren-Lawrence classification for knee OA)^22^ who underwent total knee arthroplasty. Collection of tissue samples was approved by the Medical Ethics Committee of our hospital. Participants were excluded if they had any other joint diseases (such as rheumatoid arthritis, traumatic arthritis, or gouty arthritis), any other type of macrosomia, osteochondral disease, or chronic disease (such as high blood pressure or coronary heart disease). Basic patient information is presented in Supplementary Table i. The non-diabetic group comprised five males and seven females, while the T2DM group included two males and seven females. The mean age and BMI were 68.25 years (49 to 83) and 25.13 kg/m² (22.50 to 29.62) for the non-diabetic group, and 69.44 years (59 to 75) and 26.09 kg/m² (20.81 to 28.34) for the T2DM group, with neither group meeting obesity criteria (BMI > 28 kg/m²). Although the T2DM group exhibited a slightly higher BMI, this difference was not statistically significant (p = 0.164, Mann-Whitney U test; Supplementary Figure 1A), possibly due to the limited sample size. Subsequent multiomics analyses were conducted within this cohort of 21 patients.

### Haematoxylin and eosin staining

Briefly, a portion of the tissues designated for scRNA-seq fixed, dehydrated, and paraffin-embedded. Sequential 5 μm sections were then cut, stained with haematoxylin for five minutes, rinsed, immersed in ammonia, rinsed again, and counterstained with 1% eosin. Finally, all slides were digitally scanned and analyzed using a fully automated slide scanning system (Aperio VERSA 8; Leica, Germany). Supplementary Figure 1B presents representative haematoxylin and eosin (H&E) staining of synovium and cartilage tissues processed for scRNA-seq.

### Single-cell isolation and library construction

Cartilage tissue from the damaged areas (medial condyle) and the surrounding synovial tissue were collected for subsequent experiments. Four pairs of samples for single-cell RNA sequencing were immediately used for single-cell suspension preparation, and the remaining samples were stored at -80°C before bulk RNA sequencing and metabolome assay. After acquisition, fresh cartilage and synovial tissue were washed thrice with sterile phosphate-buffered saline (PBS). The cartilage and synovial tissue were minced into 1 mm^3^ pieces and digested with 2 mg/ml collagenase II (Invitrogen; Thermo Fisher Scientific, USA) diluted in Dulbecco’s modified eagle medium/nutrient mixture F-12 (DMEM/F12) solution at 37°C for 15 hours and two hours, respectively. Undigested tissue was removed by filtration through a 70 μm cell strainer. Cell suspensions were examined under a microscope after staining with 0.4% trypan blue. When the viability of the cells was higher than 80%, library construction was performed following the 10 x Genomics protocol (10x Genomics, USA). Briefly, single cells, reagents, and gel beads containing barcoded oligonucleotides were encapsulated into nanoliter-sized gel bead-in-emulsion (GEMs) using GemCode Technology (10x Genomics). Lysis and barcoded reverse transcription of polyadenylated messenger RNA (mRNA) from single cells were performed inside each GEM. Post reverse transcription-GEMs (RT-GEMs) were cleaned up and complementary DNA (cDNA) was amplified. cDNA was fragmented and fragment ends were repaired. A-tailing was added to the 3’ end. The adaptors were ligated to the double-sided solid phase reversible immobilization (SPRI) fragments. Another double-sided SPRI selection was performed after the sample index polymerase chain reaction (PCR). The final library was qualified and quantified using two methods: checking the distribution of the fragment size using an Agilent 2100 bioanalyzer and quantifying the library using real-time quantitative PCR (qPCR; TaqMan Probe, Thermo Fisher Scientific). The final products were sequenced using the DNBSEQTM platform at BGI (BGI, China).

### Processing and analysis of single-cell RNA sequencing data

Cell Ranger (v.6.1.2, 10x Genomics) was used to demultiplex reads using the standard pipeline of 10x Genomics, followed by the extraction of cell barcodes and unique molecular identifiers (UMIs). The cDNA insert was aligned to the reference human genome (GRCh38-2020-A). After acquisition of raw gene expression matrices, the output data were processed using the Seurat package (v.4.4.0) in R software (v. 4.3.1, R Foundation for Statistical Computing, Austria). Low-quality cells were filtered by removing cells with the number of expressed genes (> 8,000 and < 500), total counts (< 500), or percentage of mitochondrial genes > 15%. The DecontX package (v.1.0.0) was used to decontaminate ambient RNA in each sample.^23^ Cells identified as doublets were removed using the DoubletFinder package (v.2.0.3).^24^

After quality control, the data were integrated, and batch effects were removed using Harmony (v.0.1.1) pipeline with principal components (PCs) = 30.^25^ The clustering analysis was performed on the integrated objects by using the “FindNeighbors” (dims = 1:30) and “FindClusters” (resolution = 2.7 in cartilage and 0.4 in synovium) functions in the Seurat package. A total of 35 clusters were identified in the cartilage (Supplementary Figure 2A, Supplementary Table ii) and 17 in the synovium (Supplementary Figure 2B, Supplementary Table iii). The identified clusters were visualized and produced using the t-distributed stochastic neighbour embedding (t-SNE) method. The “FindAllMarkers” function in the Seurat package was applied to identify the differential expressed marker genes in each subpopulation using the Mann-Whitney U test (adjusted p-value < 0.05, only.pos= T, logfc.threshold = 0.5, min.pct = 0.1). These cluster-specific genes were used to enrich the gene ontology (GO) biological process (BP) terms using the clusterProfiler package (v.4.8.2, Southern Medical University, China). The identity of each cluster was annotated based on known marker genes in previously published articles,^13,26-35^ and the clusters were considered to be the same subpopulation based on shared marker genes. The UCell score was calculated to prove the accuracy of the annotations using the “AddModuleScore” function in Seurat. In the synovium, four clusters (clusters 13 to 17, Supplementary Table iii) expressed specific markers for two or more known cell types that were identified as doublets and were removed.

Differential abundance testing between T2DMOA and OA was performed using the MiloR package (v.0.99.12) following the standard pipeline.^36^ For k-nearest neighbour graph construction, we used our reduced dimension output from the principal component analysis (PCA). For differential abundance testing, we compared T2DMOA versus OA (testNhoods, MiloR package).

The R packages VECTOR (v.0.0.3) and Slingshot (v.2.8.0) were used to perform trajectory inference analysis on the scRNA data of Fib using the standard pipeline.^37,38^

### Bulk RNA sequencing

In total, 33 samples (15 cartilage and 18 synovial tissues) were used for bulk RNA sequencing (Supplementary Figure 1C). Total RNA from the cartilage and synovial tissues was extracted using TRIzol reagent (Thermo Fisher Scientific). After quality control of the samples, mRNA libraries were prepared as previously described,^39^ and sequenced on the DNBSEQ platform at BGI (BGI, China). The raw data were filtered using SOAPnuke (v.1.6.5).^40^ The clean data were then mapped to the reference genome using HISAT (v.2.2.1)^41^ and to the assembled unique gene using Bowtie2 (v.2.4.5).^42^ Gene expression levels were calculated using RSEM software (v. 1.3.1, Dewey).^43^

### Deconvolution analysis

Deconvolution analysis was performed using the BayesPrism package (v.2.1.1, Merotto) to integrate the scRNA-seq and bulk RNA-seq data to explore the composition of synovial cells in patients with OA and T2DMOA.^44^ Deconvolution analysis was processed following the standard pipeline using protein-coding genes. Finally, the Mann-Whitney U test was performed to determine whether cell proportions were significantly different between patients with OA and T2DMOA or not.

### Functional enrichment analysis

The differential pathways between T2DMOA and OA in both bulk RNA-seq data of cartilage and synovium were identified by gene set enrichment analysis (GSEA) using the GSEA software (v.4.3.2, Broad Institute, USA). The hallmark gene sets in the MSigDB database (MSigDb and GSEA, USA) were used for the analysis, and gene set permutations were performed 1,000 times for each analysis. We considered a pathway with an false discovery rate (FDR) p-value < 0.05 as a significantly enriched pathway. The differential GO BP terms were also identified between T2DMOA and OA in scRNA-seq data of cartilage and synovial tissues using clusterProfiler package on genes detected by “FindMarkers” function of Seurat (adjusted p-value < 0.05, only.pos = F, logfc.threshold = 0.25, min.pct = 0.1) with a differential expression (DE) score calculated as previously described.^45^ The DE score represents the ratio of (upregulated genes minus downregulated genes) to the total number of enriched genes within a pathway, based on enrichment analysis conducted with cell-type-specific differentially expressed genes (DEGs). Up-regulated DEGs in lining fibroblast (Lin Fib) and sublining fibroblast (Sub Fib) between T2DMOA and OA were also identified using “FindMarkers” (adjusted p-value < 0.05, only.pos = T, logfc.threshold = 0.5, min.pct = 0.1), and their enriched GO BP pathways were detected to draw the radar map. We considered a pathway with an adjusted p-value < 0.05 as a significantly enriched pathway.

### Cellular communication analysis

Cell-cell communication analysis was performed using the CellChat package (v.2.1.1) for patients with OA and T2DMOA. When analyzing the cell-cell communication within cartilage or synovial tissue, we used default parameters and a full human ligand-receptor database (“Secreted Signaling”, “ECM-Receptor”, “Cell-Cell Contact”, and “Non-protein Signaling”).^46^ However, when focusing on ligand-receptor interactions between chondrocytes and synovial cells, only “Secreted Signaling” was included. A dot plot was used to show the difference in interaction strength for selected ligand-receptor pairs between T2DMOA and OA tissues, as previously described.

### Immunohistochemistry staining

Knee synovium and cartilage tissue sections were deparaffined in xylene and hydrated in gradient ethanol. After washing with PBS three times, sections were incubated with 3% H_2_O_2_ for 15 minutes to block endogenous catalase. Then tissue sections were incubated with prewarmed 1% trypsin (HYCEZMBIO, China) at 37°C for 30 minutes for antigen retrieval. After rinsing, sections were sealed with goat serum (Servicebio, China) for 20 minutes at room temperature and incubated with rabbit anti-tumour necrosis factor ligand superfamily member 12 (TNFSF12; 1:500, GB111772-100, Servicebio), rabbit anti-tumour necrosis factor receptor superfamily member 12A (TNFRSF12A; 1:100, 19836-1-AP, Proteintech, China), rabbit anti-hepatocyte growth factor (HGF; 1:100, 26881-1-AP, Proteintech), rabbit anti-mesenchymal-epithelial transition (MET; 1:500, 25869-1-AP, Proteintech), rabbit anti-fibroblast growth factors (FGF) 10 (1:250, PK88259; Abmart, China), rabbit anti-FGF receptor 1 (FGFR11:200, BA0485; Boster, China), rabbit anti-nicotinamide phosphoribosyltransferase (NAMPT) (1:400, GB113478-100, Servicebio), rabbit anti-ITGA5 (1:300, 10569-1-AP, Proteintech), rabbit anti-ITGB1 (1:300, GB115173-100, Servicebio), rabbit anti-angiopoietin-like protein 2 (ANGPTL2; 1:50, 12316-1-AP, Proteintech), rabbit anti-toll-like recptor 4 (TLR4; 1:300, GB11519-100, Servicebio), rabbit anti-atypical chemokine receptor 1 (ACKR1; 1:20, 55185-1-AP, Proteintech), rabbit anti-C-X-C motif chemokine ligand 8 (CXCL8; 1:50, 27095-1-AP, Proteintech) overnight at 4°C. After staining with 3,3'-diaminobenzidine (DAB) and counterstaining with haematoxylin, tissue sections were dehydrated in gradient ethanol (50%, 60%, 70%, 80%, 90%, and 95%) and sealed with neutral balsam mounting medium. All of the images were captured using a fully automated scanner (Pannoramic MIDI II, 3DHISTECH, Hungary). ImageJ 1.8.0 software (National Institutes of Health, USA) was used to analyze the immunohistochemistry (IHC) results. The staining intensity was determined by measuring the average optical density (AOD) in ten different fields for each sample. GraphPad Prism (GraphPad Software, USA, v. 9.0) was used for data analyses. All data values shown are presented as the means (standard errors of the mean (SEM)). We used the Mann-Whitney U test for comparisons between the OA and T2DMOA groups. p < 0.05 was considered statistically significant.

### Transcription factor analysis

The transcription factor analysis was performed using pySCENIC (v.0.12.1) on Python (v.3.7, Python Software Foundation, USA) for constructing gene regulatory networks from scRNA-seq data.^47,48^ To improve the computational efficiency, 2,000 cells were extracted from each sample for analysis using the “downsample” function in Seurat. Following the standard pipeline, the normal and binary regulon activity matrices and significantly enriched transcription factors (TFs) and their direct target genes were obtained. In addition, the cell-type specificity score was calculated for each subpopulation as previously described,^49^ and the essential regulators were predicted as those associated with the highest cell type-specific scores.

Regulon modules were identified based on the Connection Specificity Index (CSI),^50,51^ which is a context-dependent measure for identifying specific associating partners. Hierarchical clustering with Euclidean distance was performed based on the CSI matrix to identify different regulon modules in both the synovial and cartilage tissues. We used CSI > 0.5 as a cutoff to build the regulon association network to investigate the relationship between different regulons.

### Metabolome of articular tissues and metabolic pathway analysis

A total of 36 samples (18 cartilage and 18 synovial tissue) were included in the metabolome assay (Supplementary Figure 1C). Liquid chromatography and electrospray ionisation-tandem mass spectrometry (LC-MS/MS) was performed for metabolite identification using Waters UPLC I-Class Plus (Waters, USA) and a tandem Q Exactive high-resolution mass spectrometer (Thermo Fisher Scientific, USA) by BGI (BGI, China). After importing the offline mass spectrometry data into Compound Discoverer (v.3.3, Thermo Fisher Scientific) software and analyzing the mass spectrometry data in combination with BMDB (BGI metabolome database, BGI, China), mzCloud database (Thermo Fisher Scientific), and ChemSpider database (Royal Society of Chemistry, UK), a data matrix containing information such as metabolite peak area and identification results was obtained. Multivariate statistical analysis and univariate analysis were used to screen diﬀerent metabolites between groups using a metabolomics analysis website from BGI. First, the overall difference between the two groups was analyzed using partial least squares discriminant analysis (PLS-DA). The variable importance of projection (VIP) value of metabolites in PLS-DA used fold change and p-value to select differential metabolites and map a volcano plot (VIP ≥ 1, fold change ≥ 1.2 or ≤ 0.83, and p < 0.05). Metabolic pathway enrichment analysis of the differential metabolites based on the KEGG database (Kanehisa Laboratories, Japan) was also performed. Metabolic pathways with a p-value < 0.05 were defined as the metabolic pathways with significant enrichment of differential metabolites, and the top ten metabolic pathways (less than ten, use all data) with the smallest p-value were drawn as bubble plots and network charts.

Metabolic pathway activity scores based on the KEGG database (Kanehisa Laboratories) for scRNA-seq data were calculated as previously described.^52^ The results were presented as a heatmap and violin plot. Statistically non-significant values (random permutation test, p > 0.05) are shown as blanks in the heatmap. Finally, independent-samples *t*-tests were performed to determine whether the metabolic pathway scores of all subpopulations were significantly different between the OA and T2DMOA groups. We considered a pathway with a p-value < 0.05 as a significant differential pathway.

## Results

### Single-cell transcriptional landscape of the knee cartilage

scRNA-seq was performed on eight articular cartilage specimens from the knee joints of eight donors (Supplementary Figure 1C, Supplementary Table i), resulting in 61,338 cells. After filtering the scRNA-seq data in order to exclude damaged or dead cells, ambient RNA, and putative cell doublets, 33,403 single-cell transcriptomic profiles of chondrocytes were obtained, with 13,457 originating from OA cartilage tissue and 19,946 from T2DMOA cartilage tissue (Supplementary Figure 3A). The transcriptomic profiles of chondrocytes were determined using 10x Genomics.

**Fig. 1:**
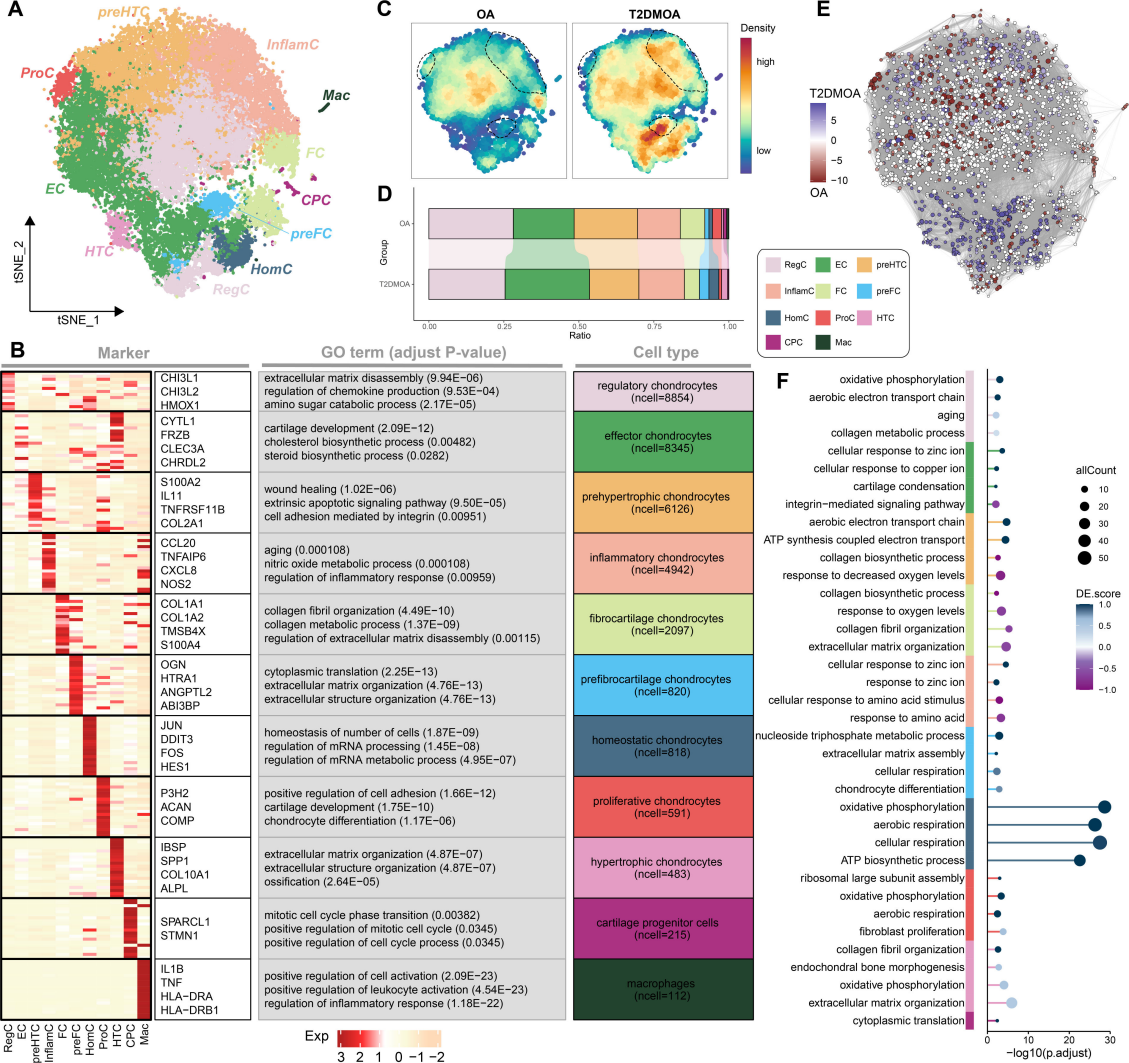
Single-cell atlas of human knee articular cartilage in patients with osteoarthritis (OA) and type 2 diabetes mellitus (T2DMOA). a) The t-distributed stochastic neighbour embedding (t-SNE) plot of the 11 identified main subpopulations in cartilage. b) Specific markers, gene ontology (GO) terms, and the number of cells for each subpopulation were visualized in a heatmap. c) Cell intensity plot and d) stacked-column chart revealing the relative proportion of each cluster across OA and T2DMOA cartilages. e) Milo analysis revealing the differential abundance of cell neighborhoods in T2DMOA (blue) compared to OA (red). f) Enriched pathways of differential expressed genes (DEGs) between T2DMOA and OA across different cell types. The colour key from white to blue (purple) indicates the absolute value of the DE score from low to high. A positive or negative DE score indicates that upregulated (blue) or downregulated (purple) DEGs comprise more than half of the enriched genes in the corresponding pathways, respectively. ATP, adenosine triphosphate; CPC, cartilage progenitor cells; EC, effector chondrocytes; FC, fibrocartilage chondrocytes; HomC, homeostatic chondrocytes; HTC, hypertrophic chondrocytes; InflamC, inflammatory chondrocytes; Mac, macrophages; preFC, prefibrocartilage chondrocytes; preHTC, prehypertrophic chondrocytes; ProC, proliferative chondrocytes; RegC, regulatory chondrocytes.

Chondrocytes were classified into 11 previously described subpopulations (Figure 1a), including regulatory chondrocytes (RegC; marked by chitinase 3 like 1 (*CHI3L1*), chitinase 3 like 2 (*CHI3L2*), and heme oxygenase 1 (*HMOX1*)), effective chondrocytes (EC; marked by *CYTL1* and *FRZB*), prehypertrophic chondrocytes (preHTC; marked by *TNFSF11B* and *COL2A1*), inflammatory chondrocytes (InflamC; marked by *CCL20* and *NOS2*), fibrocartilage chondrocytes (FC; marked by *COL1A1* and *COL1A2*), prefibrocartilage chondrocytes (preFC; marked by *HTRA1* and *OGN*), homeostatic chondrocytes (HomC; marked by *FOS* and *JUN*), proliferative chondrocytes (ProC; marked by *P3H2* and *UPP1*), hypertrophic chondrocytes (HTC; marked by *IBSP* and *COL10A1*), cartilage progenitor cells (CPC; marked by *STMN1* and *MKI67*), and macrophages (marked by *IL1B* and *TNF*), according to established markers (Supplementary Figure 3B).^13,26-29^ The specific markers, GO terms, and number of cells in each subpopulation are presented in a heat map (Figure 1b, Supplementary Tables iv and v). Interestingly, we found that two cell populations (InflamC and preFC) were notably enriched in T2DMOA chondrocytes, whereas ProCs were dominant in OA samples, according to the density and ratio of each subpopulation (Figures 1c and 1d). The results of the differential abundance of cell neighbourhoods within these three subpopulations in T2DMOA samples compared to OA samples were statistically significant using a k-nearest neighbour statistical approach,^36^ which is consistent with the above findings (Figure 1e, Supplementary Figure 3C), suggesting that these three subpopulations potentially contribute to the pathogenesis of T2DMOA.

Bulk RNA-seq was performed on the cartilage samples from 15 donors (Supplementary Figure 1C, Supplementary Table vi). GSEA of the hallmark gene set indicated that, compared to OA samples, cells in T2DMOA cartilage tissues exhibited enrichment in pathways associated with the inflammatory response and angiogenesis (Supplementary Figure 3D, Supplementary Table vii). The enriched pathways of DEGs between T2DMOA and OA across different subpopulations are shown in Figure 1f; energy metabolism-related pathways, including oxidative phosphorylation, aerobic respiration, and adenosine triphosphate (ATP) biosynthetic processes, achieved high DE scores in the majority of chondrocyte subpopulations (Supplementary Table viii).^45^ The combined results of bulk RNA-seq and scRNA-seq indicated that, compared to OA cartilage, the oxygen concentration around T2DMOA chondrocytes may change due to angiogenesis, leading to a shift in the energy metabolism pattern of chondrocytes.

### Single-cell transcriptomic atlas of the knee synovium

Eight synovial tissues paired with the above cartilage were obtained (Supplementary Figure 1C, Supplementary Table i), and the synoviocytes were profiled using scRNA-seq on a 10x Genomics platform. A total of 112,745 cells were captured, of which 78,429 cells were obtained after rigorous quality control, including doublet removal and RNA contamination removal. Of these, 38,383 cells originated from OA synovial tissues and 40,046 cells from T2DMOA tissues (Supplementary Figure da).

**Fig. 2:**
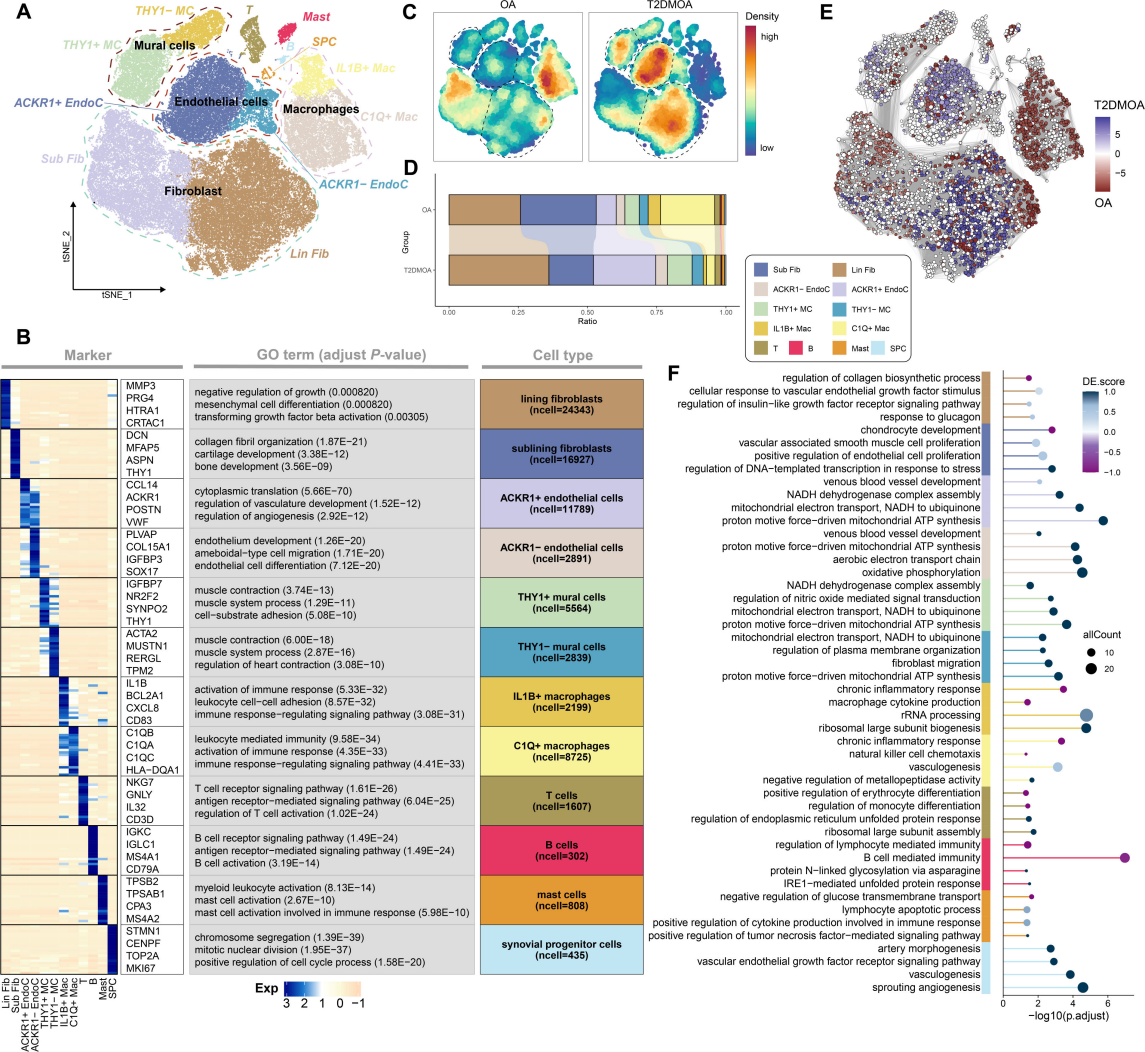
Single-cell transcriptomic analysis of osteoarthritis (OA) patients with type 2 diabetes mellitus (T2DMOA) and OA synovium. a) Visualization of clustering by t-distributed stochastic neighbour embedding (t-SNE) plot of synovial samples. The dashed lines represent the major subpopulations. b) Specific markers, gene ontology (GO) terms, and the number of cells for each subpopulation were visualized in a heatmap. c) Cell intensity plot and d) stacked-column chart revealing the relative proportion of each cluster across OA and T2DMOA synovial samples. e) Milo analysis revealing the differential abundance of cell neighborhoods in T2DMOA (blue) compared to OA (red). f) Enriched pathways of differential expressed genes (DEGs) between T2DMOA and OA across different cell types. The colour key from white to blue (purple) indicates the absolute value of the DE score from low to high. ACKR1, atypical chemokine receptor 1; ATP, adenosine triphosphate; C1Q, complement component 1 q; EndoC, endothelial cells; IL1B, interleukin 1 beta; IRE1, inositol-requiring enzyme type 1; Lin Fib, lining fibroblast; MC, mural cells; NADH, nicotinamide adenine dinucleotide; rRNA, ribosomal RNA; SPC, synovial progenitor cells; Sub Fib, sublining fibroblast.

A total of eight major subpopulations were identified, of which seven were previously reported, including fibroblasts (marked by *PDGFRA* and *PDPN*), endothelial cells (marked by *PECAM1* and *VWF*), mural cells (MC; marked by *ACTA2* and *RGS5*), macrophages (marked by cluster of differentiation (*CD) 68* and *CD163*), T cells (T; marked by *CD3D* and *NKG7*), B cells (B; marked by *CD79A* and *MS4A1*), and mast cells (Mast; marked by *TPSAB1* and *CPA3*).^30-35^ One novel subpopulation was annotated as synovial progenitor cells (SPC) based on its distinct expression of *STMN1*, *TYMS*, *PTTG1*, *PCLAF*, *CENPF*, *TOP2A*, and *MKI67*, which were associated with the cell cycle (Supplementary Figure db). Then, fibroblasts were divided into lining fibroblast (Lin Fib) and sublining fibroblast (Sub Fib), endothelial cells were classified into *ACKR1* + EndoC and *ACKR1*- EndoC, MC was divided into *THY1*+ MC and *THY1*- MC, and macrophages were annotated as *IL1B* + Mac and *C1Q* + Mac based on their respective markers (Figure 2b, Supplementary Tables ix and x). The density and ratio of each cluster are presented in Figure 2d, which were supported by the results of deconvolution using the bulk RNA-seq data from 18 synovial tissues (Supplementary Figure 1C, Supplementary Figure 5A) using BayesPrism analysis.^44^ Although all 12 subpopulations were present in both OA and T2DMOA synovial tissues, some subpopulations of major cell types, such as fibroblasts, endothelial cells, mural cells, and macrophages, appeared to be different in proportion. Fibroblasts accounted for nearly half of the total cells and did not differ between OA and T2DMOA tissues in the total population, whereas Lin Fib played a dominant role in T2DMOA, which was consistent with the differential abundance of cell neighbourhoods (Figure 2e, Supplementary Figure 5B).

The DE scores between T2DMOA and OA across different subpopulations were calculated to determine potential functional differences (Figure 2f, Supplementary Table xi). Vascular-related subpopulations, including EndoC and MC, achieved higher DE scores for energy metabolic pathways associated with aerobic respiration in T2DMOA synovial tissues than in OA tissues, indicating a possible change in oxygen concentration in the T2DMOA environment, which is consistent with the above findings in the cartilage. Compared with OA synovial tissue, Lin Fib and Sub Fib in T2DMOA tissues were down-regulated in “regulation of collagen biosynthetic process” and “chondrocyte development”, respectively, which may aggravate OA progression. In addition, the sub-Fib showed high DE scores for pathways related to cell proliferation, suggesting that DM may play a role in the proliferation and differentiation of synovial cells. A radar plot further elucidated the upregulated pathways of Lin Fib and Sub Fib (Supplementary Figure 5C). The upregulated DEGs of Lin Fib were mainly enriched in the pathways associated with vasculogenesis, while the DEGs of Sub Fib played a role in the proliferation of multiple types of synovial cells, both of which were enriched in “response to oxidative stress” and “response to reactive oxygen species”. Furthermore, the results of GSEA on the Hallmark gene set using bulk RNA-seq data (Supplementary Table xii) showed that T2DMOA samples were enriched in cell cycle-related pathways, such as the mitotic spindle and G2/M checkpoint (Supplementary Figure 5D, Supplementary Table xiii), which may also be associated with the proliferation of synovial cells. Next, we performed trajectory inference analysis to examine the differentiation relationship between the Lin Fib and Sub Fib using the VECTOR (Supplementary Figure 5E) and Slingshot (Supplementary Figure 5F) methods.^37,38^ Consistent with previous studies,^32^ Sub Fibs of the knee were initially located at the start of the trajectory and differentiated into the Lin Fib. In contrast to OA synovial tissues, more Sub Fibs differentiated into Lin Fibs in T2DMOA tissues (Supplementary Figure 5G) with the expression of genes such as *MMP1*, *MMP3*, *CXCL1*, *CXCL8*, *CLIC5*, and *PRG4* (Supplementary Figure 5H), indicating that DM might partially aggravate OA progression by inducing the accumulation of Lin Fibs.

### Intercellular signalling networks in the cartilage

CellChat analysis revealed 33 potential signalling interactions relevant to patients with T2DMOA (Figure 1) and 47 potential signalling interactions linked to OA (Supplementary Figure 6A).^46^ The global communication levels of chondrocytes showed that ProC was the strongest sender and receiver in both OA and T2DMOA cartilage tissues. Collagen and fibronectin 1 (FN1) were the top two signalling pathways among chondrocytes, which was consistent with previous studies.^29^ Moreover, cells were divided into two outgoing patterns and two incoming patterns in T2DMOA (Supplementary Figure 6B), with a synergistic effect between subpopulations within a pattern.

**Fig. 3:**
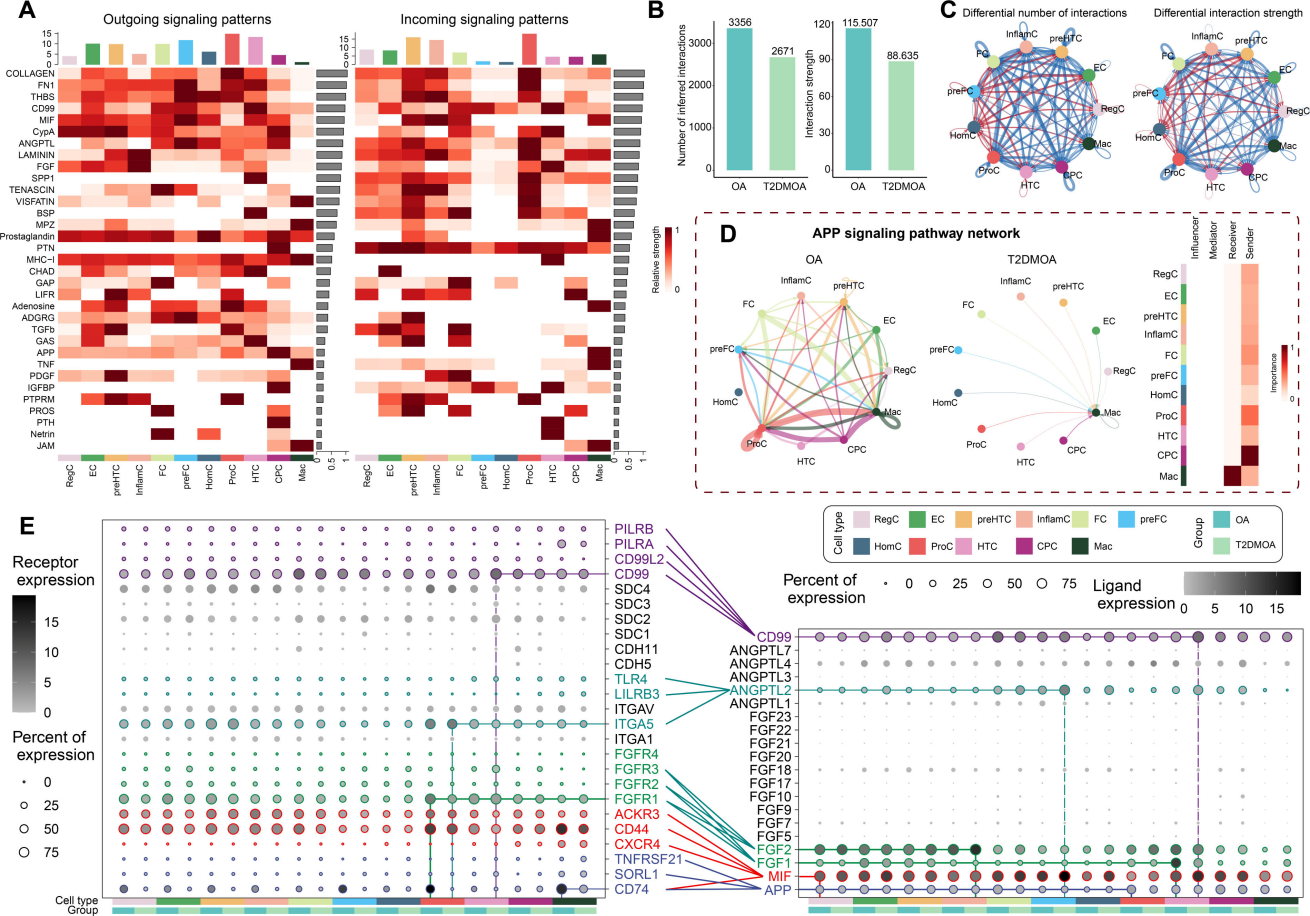
Cell-cell communications within human knee articular cartilage of patients with osteoarthritis (OA) and type 2 diabetes mellitus (T2DMOA). a) The relative strength of all enriched signals (outgoing and incoming) across T2DMOA articular cartilage clusters was visualized in a heatmap. The changes in the number and strength of cellular interactions between T2DMOA and OA are shown in a b) column chart and c) circle plot. The blue line indicates a decreased communication number/strength in the T2DMOA cartilage compared to that in the OA cartilage, while the red line indicates an increased communication number/strength in the T2DMOA cartilage. d) Circle plot showing the significant differential signalling pathways between T2DMOA and OA cartilage tissues. Heatmap showing the role of the four centrality measures in T2DMOA samples. e) Dot plot showing the difference in interaction strength for selected ligand-receptor pairs among T2DMOA and OA cartilage. Dot size indicates the percentage of ligand-receptor expression in cells of one cluster, coloured by average ligand-receptor expression levels. APP, amyloid precursor protein; CPC, cartilage progenitor cells; EC, effector chondrocytes; FC, fibrocartilage chondrocytes; HomC, homeostatic chondrocytes; HTC, hypertrophic chondrocytes; InflamC, inflammatory chondrocytes; Mac, macrophages; preFC, prefibrocartilage chondrocytes; preHTC, prehypertrophic chondrocytes; ProC, proliferative chondrocytes; RegC, regulatory chondrocytes.

The cell-cell interaction number and strength were remarkably decreased in the T2DMOA cartilage compared with those in the OA cartilage (Figures 3b and 3c). The information flow of each signalling pathway was calculated to identify the differential signalling pathways between OA and T2DMOA cartilage tissues. A total of 35 differential signalling pathways were identified, of which 30 were upregulated in OA and 5 in T2DMOA (Supplementary Figure 6C). Six signalling pathways – amyloid precursor protein (APP), migration inhibitory factor (MIF), FGF, collagen, CD99, and angiopoietin-like protein (ANGPTL) – were dominant in the information flow of the differential pathways. The APP and MIF signalling pathways were significantly attenuated in T2DMOA cartilage (Figures 3d and 3e, Supplementary Figures 7A and B). FGF signals (Figure 3e, Supplementary Figure 7C) and ANGPTL (Figure 3e, Supplementary Figure 7D) were significantly altered in the T2DMOA cartilage. Notably, the CD99 signal level was enhanced in the T2DMOA cartilage (Figure 3e, Supplementary Figure 7E). Moreover, parathyroid hormone like hormone (PTHLH), a cytokine-like peptide originating from CPC,^53^ was activated in the HTC of T2DMOA cartilage tissues (Supplementary Figure 7F). As a critical component of the matrisome, the signalling of collagen was also downregulated in T2DMOA (Supplementary Figure 7G).

### Intercellular signalling networks in the synovium

**Fig. 4:**
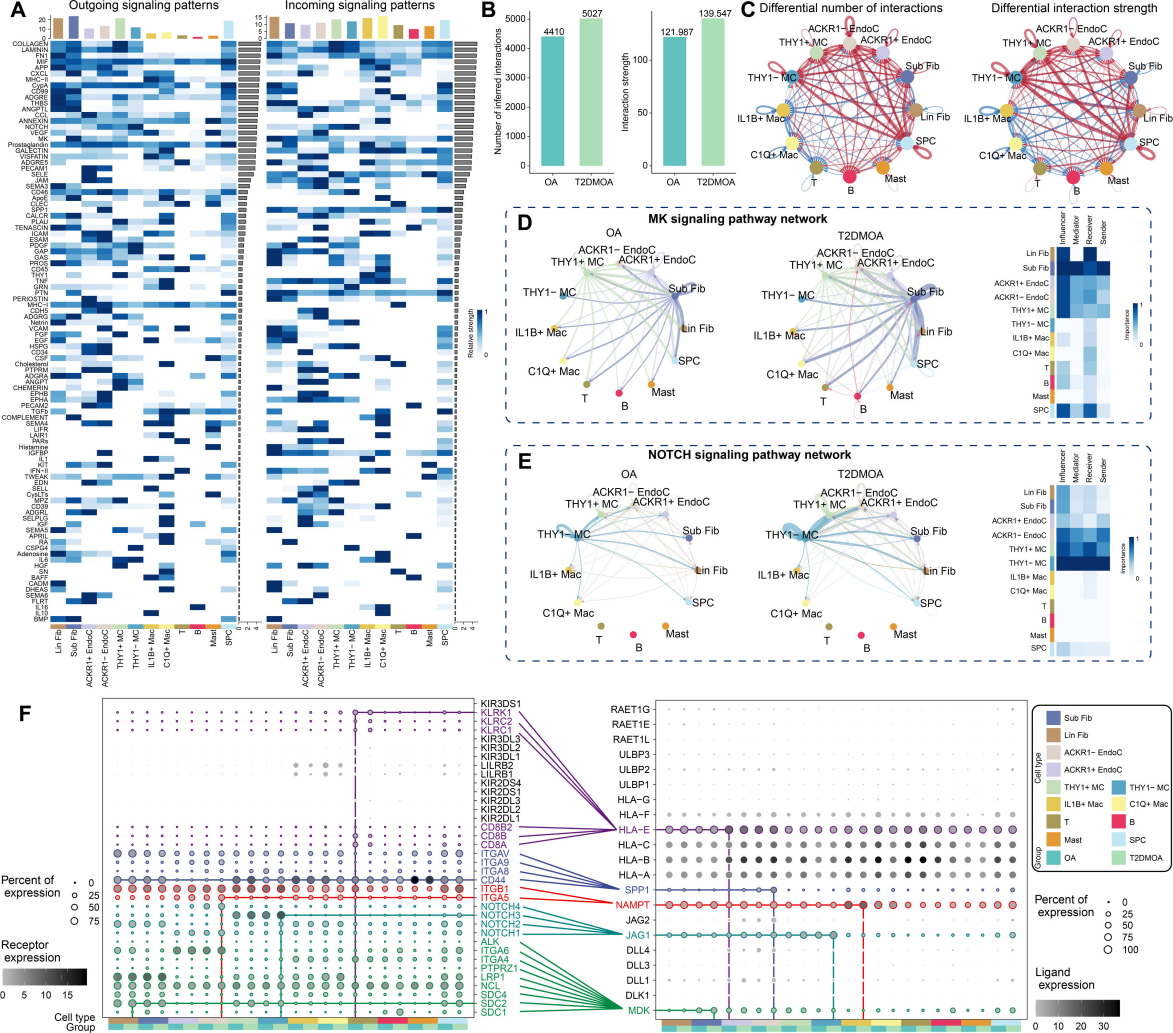
Cellular communications within human knee articular synovium of patients with osteoarthritis (OA) and type 2 diabetes mellitus (T2DMOA). a) Relative strength of all enriched signals (outgoing and incoming) across T2DMOA articular synovial clusters was visualized in a heatmap. The changes in the number and strength of cellular interactions between T2DMOA and OA are shown in a b) column chart and c) circle plot. The blue line indicates a decreased communication number/strength in the T2DMOA synovium compared to that in the OA synovium, while the red line indicates increased communication number/strength in the T2DMOA synovium. d) and e) Circle plot showing the significant differential signalling pathways between T2DMOA and OA synovial tissues, alongside a heatmap showing the role importance of the four centrality measures in T2DMOA samples. f) Dot plot showing the difference in interaction strength for selected ligand-receptor pairs among T2DMOA and OA synovium. Dot size indicates the percentage of ligand-receptor expression in cells of one cluster, coloured by average ligand-receptor expression levels. ACKR1, atypical chemokine receptor 1; EndoC, endothelial cells; IL1B, interleukin 1 beta; Lin Fib, lining fibroblast; Mac, macrophages; MC, mural cells; SPC, synovial progenitor cells; Sub Fib, sublining fibroblast.

A total of 89 overlapping signalling pathways were found between T2DMOA and OA synovial tissues (Figure 4a and Supplementary Figure 8A). In the global intercellular communication of synovial cells, Lin Fib, Sub Fib, and THY1+ MC were the strongest senders, whereas Lin Fib, IL1B + Mac, and C1Q + Mac were the strongest receivers. The top four global signalling pathways among synoviocytes in both OA and T2DMOA synovial tissues were collagen, FN1, MIF, and LAMININ. Three outgoing and four incoming communication patterns were identified to detect synergies among 12 subpopulations (Supplementary Figure 8B). The cell-cell interaction number and interaction strength were significantly increased in the T2DMOA synovium when compared to those in the OA synovium (Figures 4b and 4c), with the presence of 64 signalling pathways that were altered between the two groups and 41 pathways with no significant changes (Supplementary Figure 8C). The midkine (MK) signals, *Jagged1* (*JAG1*)-*NOTCH3* ligand receptor pair, visfatin signals, secreted phosphoprotein 1 (SPP1) signals, and major histocompatibility complex class I (MHC-I) signals were found to be significantly altered in the T2DMOA synovium compared to ordinary OA (Figures 4d to 4f, Supplementary Figures 9A to E).

### Cellular communication networks between synovium and cartilage

**Fig. 5:**
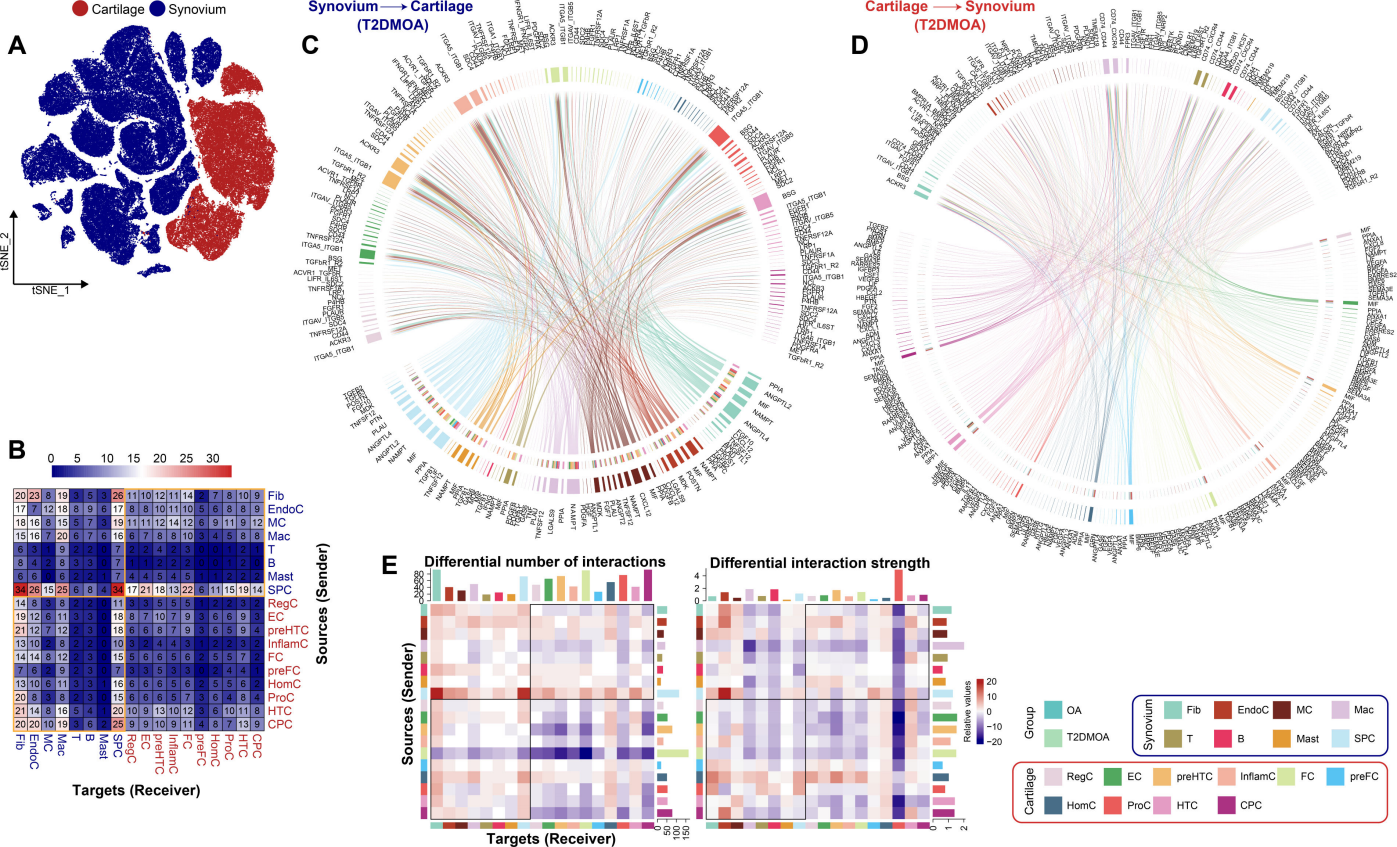
Cell-cell communication networks between human knee articular synovium and cartilage. a) Visualization of integrating synovial cells and chondrocytes by t-distributed stochastic neighbour embedding (t-SNE) plot. b) Heatmap showing the number of cell-cell interactions between synovium and cartilage in samples from patients with osteoarthritis and type 2 diabetes mellitus (T2DMOA). The number of interactions between chondrocytes and synovial cells is shown in the orange box. Chordal plot showing the ligand-receptor pairs between T2DMOA synovium and cartilage. Signals from c) synovium and d) cartilage were shown, respectively. e) Heatmap presenting difference in interaction number and strength between T2DMOA and OA tissue. The colour key from white to red (blue) indicates the relative value from low to high. CPC, cartilage progenitor cells; EC, effector chondrocytes; EndoC, endothelial cells; FC, fibrocartilage chondrocytes; Fib, fibroblast; HomC, homeostatic chondrocytes; HTC, hypertrophic chondrocytes; InflamC, inflammatory chondrocytes; Mac, macrophages; MC, mural cells; preFC, prefibrocartilage chondrocytes; preHTC, prehypertrophic chondrocytes; ProC, proliferative chondrocytes; RegC, regulatory chondrocytes; SPC, synovial progenitor cells.

CellChat analysis was performed based on both secreted and cell-surface molecules, the repository from the chondrocytes (macrophages were excluded), and major subpopulations of synovial cells encompassing ligand-receptor interactions mediated by the diffusion of secreted molecules (Figure 5a). Contrary to our prior understanding, we observed more ligand-receptor interactions between chondrocytes and synoviocytes when chondrocytes acted as sources in both T2DMOA (Figure 5b, Supplementary Table xiv) and OA (Supplementary Figure 10A, Supplementary Table xv) tissues. When synovial cells function as senders and chondrocytes as receivers, fibroblasts, endothelial cells, mural cells, macrophages, and SPCs are the main sources of signals bridged by signalling pathways that include ANGPTL, MIF, CypA, visfatin, and FGF (Figure 5c, Supplementary Figure 10B). When chondrocytes served as senders and synovial cells as receivers, all subpopulations were involved, with interactions between signalling pathways, including MIF, SPP1, FGF, CypA, and annexin (Figure 5d, Supplementary Figure 10C).

The global number and strength of interactions between synoviocytes and chondrocytes were also altered when considering the differences between OA and T2DMOA tissues. When communicating with chondrocytes, the signals from Mac and SPC were decreased and enhanced, respectively, in T2DMOA tissues. However, the ProC received significantly fewer signals from synovial cells in the T2DMOA cartilage, both in number and strength. The signals to synovial cells sent by FC were also remarkably decreased in T2DMOA tissues (Figure 5e, Supplementary Figure 10D). Alterations in the specific signalling pathways between OA and T2DMOA tissues were also detected. Seven ligand-receptor pairs (Supplementary Figures 10E and 10F, Supplementary Figures 11A to 11G) with different interaction strengths between OA and T2DMOA were selected for IHC validation in the human specimens. IHC results showed that, when compared with the OA synovium, the protein levels of HGF, FGF10, NAMPT, TLR4, ITGA5, and ITGB1 in the T2DMOA synovium were significantly increased, atypical chemokine receptor 1 (ACKR1) was decreased, and TNFSF12 showed no statistical difference (Supplementary Figure 11H). When compared to OA cartilage, the protein levels of ANGPTL2 and ITGB1 in T2DMOA cartilage were significantly increased, and TNFRSF12A, CXCL8, MET, FGFR1, and ITGA5 were not significantly different (Supplementary Figure 11H). In the signals originating from synoviocytes, except for *TNFSF12-TNFRSF12A*, alterations in the interaction strengths of *HGF-MET*, *FGF10-FGFR1*, and *NAMPT-*(*ITGA5+ ITGB1*) were consistent with those of IHC. Regarding the signalling pathways in chondrocytes, except for *CXCL8-ACKR1*, the differences seen in the interaction strength of *ANGPTL2-TLR4* and *ANGPTL2*-(*ITGA5 + IGTB1*) were consistent with the IHC results. Therefore, there is significant heterogeneity in the cellular communication between OA and T2DMOA synovial cells and chondrocytes.

### Gene regulatory networks and CSI modules of synovium and cartilage

**Fig. 6:**
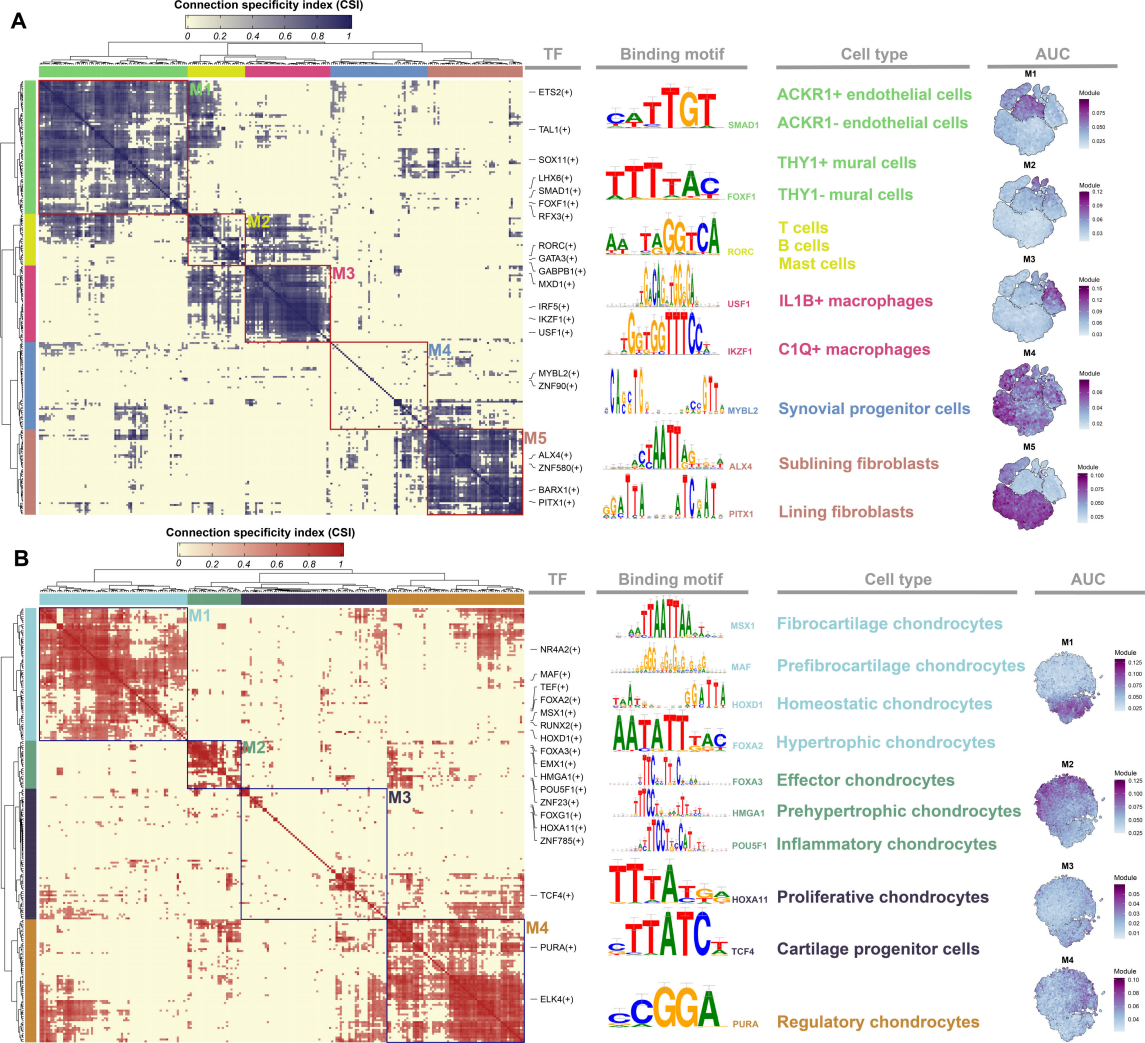
Transcription factor (TF) analysis in human knee articular synovium and cartilage of patients with osteoarthritis (OA) and type 2 diabetes mellitus (T2DMOA). Identified regulon modules in a) synovial and b) cartilage tissue based on the regulon connection specificity index (CSI) matrix, along with representative transcription factors, corresponding binding motifs, associated cell types, and average module activity scores mapped on t-distributed stochastic neighbour embedding (t-SNE). ACKR1, atypical chemokine receptor 1; AUC, area under the curve; C1Q, complement component 1 q; IL1B, interleukin 1 beta.

A total of 244 enriched regulons in synovial cells (Supplementary Table xvi) and 266 enriched regulons in chondrocytes (Supplementary Table xvii) were identified using pySCENIC analysis.^47,48^ For each regulon, we calculated the activities associated with each subpopulation in the synovium and cartilage tissues and defined a regulon specificity score (RSS). The top six regulons with the highest RSS values for each subpopulation were presented using a rank plot (Supplementary Figure 12A and B, Supplementary Tables xviii and xix), and the regulons with the highest RSS values were considered specific regulons which were used to identify cell types. Enriched TFs of synoviocytes and chondrocytes were clustered into five and four major modules, respectively (Figure 6b, Supplementary Tables xx and xxi), by comparing the similarity of regulon activity scores (RAS) (Supplementary Figures 13A and B) of each regulator pair based on the CSI.^50,51^ In synovial cells, module M1 contains specific regulons of endothelial cells and mural cells, such as *SMAD1*^54^ and *FOXF1*,^55^ which are associated with angiogenesis or proliferation of endothelial cells. Module M2 contains transcription factors *RORC*,^56^ etc., which are essential regulators of immune cells, including T, B, and Mast. Module M3 contained the regulators *IKZF1*^57^ and *USAF1*,^58^ which are specific to the polarization, inflammation, and differentiation of macrophages. Module M4 contains regulators associated with the development and proliferation of progenitor cells such as *MYBL2.*^59^ Module M5 contained specific regulons that are activated in Fib, such as LinFib and SubFib. Interestingly, regulons in M5 such as *ALX4* and *PITX1* are essential for limb development.^60,61^ Because the subpopulations of chondrocytes were more similar to each other, regulons such as *RUNX2*, *HOXA11*, and *DBP* were activated in multiple cell types. However, some unique TFs that were activated in the subpopulations also divided the regulators into four modules. Module M1 contained regulators associated with bone formation and chondrocyte hypertrophy, such as *MSX1*,^62^*MAF*,^63^ and *FOXA2*.^64^ Regulons in M2 are highly associated with chondrocyte differentiation, such as *FOXA3*,^65,66^*HMGA1*,^67^ and *POU5F1*.^68^ Module M3 contains specific skeletal stem cell factors such as *HOXA11.*^69^ Regulators in M4 are highly associated with RegCs. Interestingly, the regulons clustered in the same module appeared to have similar binding motifs.

A t-SNE map was used to display the average activity score of each module. We found that each module occupied a different region and could map to the corresponding subpopulations, except for the M4 module in synovial tissue, which mapped to almost all regions and might be associated with the function of SPC. Therefore, related subpopulations appear to share a similar overall network structure (Supplementary Figures 13C and D). These analyses predicted the critical regulators of each subpopulation in the synovial and cartilage tissues of the knee. A sample-specific regulon analysis based on binarized RAS was also performed to further explore the differences in regulons between OA and T2DMOA synovial tissues and cartilage. The average RAS values for each sample were converted to zero and one to determine whether the TFs were activated. Regulons were identified as sample-specific only if they were activated in two or more samples, and in one or fewer samples from another group. However, a regulon that was activated in at least six of the eight samples was considered a common TF. The results showed that there were two specifically activated TFs (*MEIS1* and *NR2F6*) in T2DMOA synovial tissue (Supplementary Figure 13E) and three (*MAF*, *MBD2*, and *POU4F3*) in T2DMOA cartilage tissue (Supplementary Figure 13F). To identify the target genes regulated by the sample-specific regulons, regulons with area under curve (AUC) values greater than 0.1, including *NR2F6* in synovial tissues and *MAF* in cartilage tissues, were selected for subsequent analyses. The results were presented as a TF-target network plot (Supplementary Figures 13G and H).

### Alterations in metabolic pathways of synovium and cartilage

**Fig. 7:**
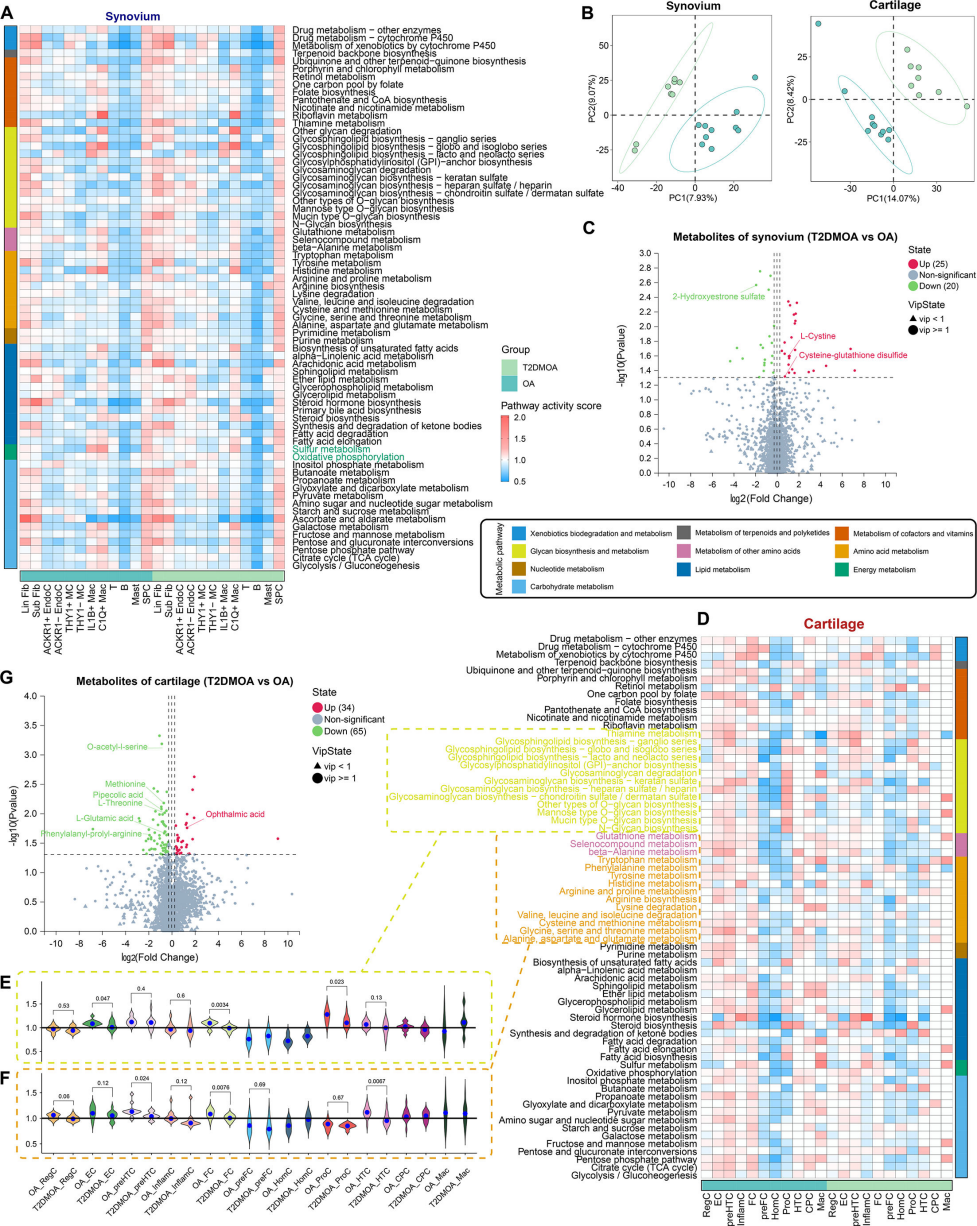
Alterations in metabolic pathways of human knee synovium and cartilage between patients with osteoarthritis (OA) and type 2 diabetes mellitus (T2DMOA). a) Metabolic pathway activity in subpopulations in the single-cell RNA sequencing (scRNA-seq) dataset of synovium. A pathway activity score > 1 (red) indicates activation of the pathway. Statistically non-significant values (random permutation test p > 0.05) are shown as blank. b) Partial least squares discriminant analysis (PLS-DA) analysis showing the relationship between metabolite expression and sample category in articular synovium and cartilage. c) Volcano plot showing differential metabolites in synovium between T2DMOA and OA tissue. The red dots represent upregulated metabolites and the green dots represent down-regulated metabolites. Metabolites of interest are labeled in the plot. d) Metabolic pathway activities in subpopulations in the scRNA-seq dataset of cartilage. e) and f) Violin plot showing the difference in metabolic pathway activity score of e) glycan biosynthesis and metabolism and f) amino acid metabolism + metabolism of other amino acid between T2DMOA and OA cartilage. g) Volcano plot showing differential metabolites in cartilage between T2DMOA and OA tissue.

In synovial cells, most metabolic pathways were activated in Lin Fib, Sub Fib, and SPC, but were inhibited in T, B, and Mast (Figure 7a, Supplementary Figure 14A). Interestingly, the number of pathways in the energy metabolism category activated in T2DMOA synovial cells (pathway activity score > 1) was greater than that in OA cells (Figure 7a, Supplementary Table xxii), especially in Lin Fib, Sub Fib, *THY1 +* MC, and *THY1-* MC, which is consistent with the results shown in Figure 2f. To further validate our findings, we applied metabolic mass profiles based on liquid chromatography and electrospray ionization tandem mass spectrometry (LC-MS/MS) to 18 synovial samples from ten patients with OA and eight with T2DMOA (Supplementary Figure 1C, Supplementary Table i). Partial least-squares discriminant analysis (PLS-DA) was performed on the two groups of biological samples to establish the relationship between the expression of metabolites and each group of samples. Samples of T2DMOA and OA were separated (Figure 7b), and 45 differential metabolites were detected, of which 25 were upregulated in T2DMOA synovial tissues (Figure 7c, Supplementary Table xxiii). Metabolites such as methylpyrogallol sulphate 2, L-cysteine, and cysteine-glutathione disulfide were significantly upregulated in the T2DMOA synovium, and 2-hydroxyestrone sulfate was downregulated in the T2DMOA synovium, suggesting that the sulphur metabolism pathway and metabolites were altered in the T2DMOA synovium compared to those in OA.

In the cartilage, EC, prehypertrophic chondrocytes (preHTCs), and FC showed high metabolic activity. However, unlike synovial tissues, almost all subpopulations in cartilage tissues showed reduced metabolic pathway scores in T2DMOA tissues (Figure 7d, Supplementary Figure 14B). Proteoglycans and collagen are essential components of the extracellular matrix (ECM) of chondrocyte ECM. However, the activities of pathways, including glycan biosynthesis and metabolism (Figure 7e), amino acid metabolism, and metabolism of other amino acids (Figure 7f), in most T2DMOA subpopulations, especially preHTC and HTC which are the main cell types that synthesize ECM, were remarkably downregulated compared with those in OA (Supplementary Table xxiv). The metabolome profiles of OA and T2DMOA cartilage tissues were also analyzed (Supplementary Figure 1C), and the two groups were separated (Figure 7). Compared to OA, 34 metabolites were upregulated and 65 were downregulated in T2DMOA cartilage, including a variety of amino acids such as O-acetyl-l-serine, methionine, pipecolic acid, L-threonine, and L-glutamic acid (Figure 7g, Supplementary Table xxv). Notably, these downregulated metabolites were enriched in pathways associated with amino acid metabolism and transport, including cysteine and methionine metabolism, amino acid biosynthesis, arginine and proline metabolism, aminoacyl-tRNA biosynthesis, and ABC transporters (Supplementary Figure 14C, Supplementary Table xxvi), which was consistent with the results of the scRNA-seq data. Pathway enrichment network analysis of the differential metabolites based on the KEGG database showed that l-glutamic acid is involved in multiple metabolic pathways and may be a key metabolite in T2DMOA pathology (Supplementary Figure 14D).

## Discussion

In the past decade, metabolic features of OA have become increasingly visible, leading to the definition of T2DMOA as a novel subtype of OA, and brought changes to the diagnosis and treatment of OA that deviated from traditional concepts.^44^ Multiple links have been discovered between metabolic OA and metabolic syndrome, either with metabolic syndrome as a whole, or its components like obesity, DM, hyperlipidemia, and hypertension.^70^ We have previously reported that hyperglycaemia could cause the accumulation of advanced glycosylation end products in the synovium, thereby stimulating the release of inflammatory factors from fibroblast-like synoviocytes and aggravating the progression of OA.^11^ Li et al^71^ reported that the timely administration of metformin, the most widely applied antidiabetic agent worldwide, could attenuate the development and progression of post-traumatic OA in an animal model of destabilization of the medial meniscus. Such findings indicated that metabolic factors play critical roles in OA progression, and that targeting the pathogenesis of DM could be a novel strategy for OA treatment.

A previous study has classified chondrocyte and synoviocyte phenotypes, and reconstructed cell-type lineage-recapitulating factors and multiple signalling pathways that contribute to OA progression.^72^ However, the effects of metabolic factors, especially DM, on these processes remain unclear. InflamC and preFC were notably enriched in T2DMOA chondrocytes, while ProCs were dominant in OA samples in the 11 subpopulations identified in our current study, which indicated that DM promoted local inflammation, fibrosis, and inhibited cell proliferation of the cartilage, resulting in an advanced stage of OA. In response to alterations in the joint microenvironment, chondrocytes may undergo metabolic reprogramming, a shift that can contribute to cartilage degeneration and the progression of OA.^73^ The energy metabolism of normal chondrocytes mainly relies on glycolysis due to the hypoxic environment of the cartilage, while pyruvate oxidation and tricarboxylic acid cycle are enhanced in the early stage of OA progression.^74^ Here, energy metabolism-related pathways, including oxidative phosphorylation, aerobic respiration, and ATP biosynthetic process were stimulated in T2DMOA cartilage, indicating a metabolic reprogramming of the chondrocyte from anaerobic metabolism to aerobic metabolism in the T2DMOA progression when compared to ordinary OA. Such changes were consistent with the changes observed in the synovium, namely that energy metabolic pathways associated with aerobic respiration and vasculogenesis were enriched in the T2DMOA samples, indicating that the rise of oxygen concentration in the joint and the metabolic reprogramming of both the cartilage and synovium might be the key processes in the pathogenesis of T2DMOA.

Intercellular signalling networks among subpopulations of chondrocytes and synoviocytes were investigated in the current study, as well as those between the synovium and articular cartilage based on ligand-receptor interacting pairs in patients with OA or T2DMOA. We found that signals, including *HGF-MET*, *FGF10-FGFR1*, and *NAMPT-*(*ITGA5 + ITGB1*) from synoviocytes to chondrocytes, as well as *ANGPTL2-TLR4* and *ANGPTL2-*(*ITGA5 + IGTB1*) from chondrocytes to synoviocytes, showed notable alterations in the results of both scRNA-seq and IHC from patients with T2DMOA. Therein, *HGF-MET*^75^ and *FGF10-FGFR1*^76^ pairs promotes bone destruction in OA, and the visfatin pathway may contribute to osteoarthritic cartilage destruction.^77^ One previous study has shown that ANGPTL2 activated the NF-κB and p38/MAPK signalling pathways via ITGA5 and ITGB1 and accelerated cartilage degeneration.^78^ These ligand-receptor interacting pairs are quite different from those in the previous study based on OA and non-OA synovium and articular cartilage,^72^ which indicated that the above signals might be the typical signals for the pathogenesis of T2DMOA.

Our knowledge of cellular heterogeneity has increased over the past few years. In comparison, for most cell types identified thus far, we lack a mechanistic understanding of how their characteristic gene expression programs are established and maintained. In this study, we comprehensively constructed gene regulatory networks for all major cell types in cartilage and synovial tissues through computational analysis. An important contribution of this study is the prediction of the critical regulators for each cell type. Although most of the predictions are hypotheses, they provide guidance for future experimental investigations. Moreover, sample-specific regulon analysis based on binarized RAS was performed to identify T2DMOA-specific TFs. *NR2F6*, a critical trans-regulator in oncogenesis, immunity, and metabolic regulation, was shown to be a specific regulon of Fib in T2DMOA synovium that regulated 13 target genes.^79,80^ In addition, *MAF* is specific to both T2DMOA chondrocytes and preFC, which regulates ten target genes.

In the last decade, multiomics analyses have provided crucial new insights into the interplay between both the intracellular and intercellular molecular mechanisms involved in disease pathogenesis.^81,82^ Our findings have shown that most metabolic pathways were activated in the Lin Fib, Sub Fib, and SPC in the T2DMOA synovium. The number of pathways in the energy metabolism category that were activated in T2DMOA synovial cells was greater than that in OA cells, particularly in the Lin Fib and Sub Fib groups. In cartilage, EC, preHTCs, and FC presented high metabolic activity, whereas in most T2DMOA subpopulations, especially preHTCs and HTC, metabolic activity was significantly reduced compared to OA. These cells are the main cell types that synthesize the ECM. Meanwhile, the activities of pathways, including glycan biosynthesis and metabolism, amino acid metabolism, and the metabolism of other amino acids, were remarkably downregulated in T2DMOA samples when compared to ordinary OA. These changes were highly consistent with the metabolic shifts observed in both chondrocytes and synoviocytes in T2DMOA. Further metabolomic data showed that the sulphur metabolism pathway and its metabolites were significantly altered in the T2DMOA synovium, whereas the activities of pathways associated with amino acid and glycan metabolism were notably downregulated in the T2DMOA cartilage. O-acetyl-l-serine, methionine, pipecolic acid, L-threonine, L-glutamic acid, and other amino acids were significantly decreased in T2DMOA cartilage. L-glutamic acid, an essential amino acid and a key player in the tricarboxylic acid cycle,^83^ has been shown to be involved in multiple metabolic pathways in T2DMOA chondrocytes, which again indicates that DM induces the metabolic reprogramming of chondrocytes, and L-glutamic acid may be a key metabolite in T2DMOA pathology.

In conclusion, the current study has provided insights into the transcriptional landscape, intercellular signalling networks, cellular metabolic alterations, and gene regulatory networks in the pathogenesis of T2DMOA and revealed that metabolic reprogramming was induced in both chondrocytes and synoviocytes, which adds novel evidence to the treatment of T2DMOA.

## Data Availability

The data that support the findings for this study are available to other researchers from the corresponding author upon reasonable request.
